# Carbohydrate Mouth-Rinsing Improves Overtime Physical Performance in Male Ice Hockey Players During On-Ice Scrimmages

**DOI:** 10.3389/fnut.2022.792708

**Published:** 2022-02-10

**Authors:** Danielle L. E. Nyman, Alexander S. D. Gamble, Jessica L. Bigg, Logan A. Boyd, Alexander J. Vanderheyden, Lawrence L. Spriet

**Affiliations:** ^1^Department of Human Health and Nutritional Sciences, University of Guelph, Guelph, ON, Canada; ^2^School of Kinesiology and Health Studies, Queen's University, Kingston, ON, Canada

**Keywords:** team sport, local positioning system, heart rate monitors, external load, internal load, 3-on-3 scrimmage, hydration

## Abstract

**Purpose:**

This randomized, double-blind, crossover study examined the effects of mouth-rinsing (MR) with a carbohydrate (CHO) vs. a placebo (PLA) solution on external and internal loads in hydrated ice hockey players during regulation and overtime (OT) periods of an on-ice scrimmage.

**Methods:**

Twelve skilled male hockey players (22.6 [3.4] years, 178.9 [4.7] cm, 84.0 [6.5] kg) played three 20-min regulation periods and one 12-min OT period of small-sided 3-on-3 scrimmage. Skaters repeated 2 min shift and rest intervals. Participants mouth rinsed with 25 mL of CHO or PLA solution approximately every 10 min for a total of 7 rinses. A local positioning system (LPS) tracked external load variables including speed, distance, acceleration, and deceleration. Internal load was monitored with heart rate (HR) sensors and a rating of perceived exertion (RPE).

**Results:**

During regulation play, both the conditions developed similar fatigue, with significantly decreased high-intensity distance, average speed and decelerations, and increased RPE, from period 1 to 2 and 3. In OT, CHO MR increased the distance skated at high-intensity (224 [77], 185 [66] m, *p* = 0.042), peak speed (24.6 [1.6], 23.7 [1.3] km·h^−1^, *p* = 0.016), number of sprints (1.9 [1.2], 1.2 [0.9], *p* = 0.011), and decreased distance skated at slow speed (300 [33], 336 [47], *p* = 0.034) vs. PLA MR. OT RPE was similar between the two conditions in spite of more work done in CHO MR.

**Conclusions:**

CHO MR may be a valuable practice to protect against decrements in external load with increased playing time in ice hockey, and possibly allows athletes to perform more work relative to perceived levels of exertion.

## Introduction

Ice hockey is an intermittent high-intensity team sport that requires explosive power, speed, muscular strength, and superior anaerobic and aerobic capacities (1–6). On-ice shifts are short (30–80 s), but players perform several bouts of near maximal-intensity exercise per minute, including repeated sprints, quick direction changes, body contact, grappling, and rapid accelerations and decelerations (1–5). These bouts are interspersed with periods of low-intensity skating and gliding, and rest.

Given the high physical intensity of training and competition, carbohydrate (CHO) from muscle glycogen is the primary fuel for ice hockey (3, 7, 8). Historical research has established that intermittent periods of high-intensity skating significantly exacerbated skeletal muscle glycogen depletion relative to continuous steady-state skating (9, 10), and subsequent work confirmed significant depletion of both type I and type II muscle fibers from pre- to postgame in male hockey players (7, 11, 12). Providing greater details to the limited pool of on-ice research, Vigh-Larsen et al. recently established time-point analysis of muscle glycogen depletion and the development of fatigue in elite men (U20 International) during a controlled hockey game (3). Despite playing only 8, 1-min shifts per period, significant glycogen depletion occurred within period 1, and ~65% of fast- and slow-twitch fibers were depleted by the end of the 3 periods. These findings were associated with marked decrements in physical performance across time, including reduced repeated sprint ability and fewer accelerations and decelerations, which were attributed to the development of fatigue. The glycogen depletion and fatigue exhibited by hockey players are similar to postgame values reported in field sport athletes, but notably, are achieved with dramatically less playing time (24 vs. ~90 min) (13). From the collective findings of ice hockey and other intermittent high-intensity exercise research, it has been established that low energetic states within skeletal muscle fibers produce intolerance to repeated high-intensity exercise bouts and exacerbate fatigue (3, 13, 14).

In previous research, it was established that the consumption of CHO electrolyte solutions (CES) improved physical performance and helped reduce fatigue across time in ice hockey (15–17). This was observed as increased voluntary work performed, skating speed and time at high effort, and reduced ratings of perceived exertion (RPE) with CES ingestion. However, these results were compared to no-fluid, mild dehydration trials, and so it remains unclear whether performance enhancements were the result of mitigation of dehydration, CHO ingestion, oral exposure to CHO, or a combination of these factors.

Indeed, isolated oral exposure to CHO through mouth-rinsing (MR) has demonstrated small to moderate positive effects on exercise performance in short-term (< 60 min), steady-state high-intensity (> 75% VO_2_max) running and cycling. Relative to taste-matched placebo solutions (PLA), CHO MR was observed to improve time trial (TT) performance and power production, as well as increase distances and times to exhaustion (18–20). In seminal work, direct infusion of glucose did not enhance exercise performance, despite elevated blood glucose levels and increased muscle glucose uptake (21). Thus, it was speculated that the mechanism responsible for performance improvement with CHO MR may be non-metabolic and related to central control. To test this hypothesis, Chambers et al. conducted a multipart study that examined human brain activity and exercise performance in response to isolated oral exposure to isocaloric sweet (glucose) and tasteless CHO (maltodextrin), as well as a non-caloric sweetener (saccharin) (22). MR with either CHO solution improved performance relative to the non-caloric sweet placebo. Notably, both CHO solutions, but not the PLA solution, increased brain activity in the reward, motivation, and motor control regions, independent of taste. Similar findings have been repeated with other non-caloric sweeteners (ex. sucralose), which also demonstrated a minimal effect on brain activity in these regions (23). Building on the seminal findings, later works theorized that oral sensing of CHO induces subconscious perceptions of energy availability that alter central control, and demonstrated the facilitation of increased corticomotor and sensorimotor activity with CHO MR, which are proposed to mitigate fatigue and attenuate performance decrements (24–26).

Though CHO MR has been thoroughly examined in steady-state exercise, there is mixed reporting of potential benefits in intermittent high-intensity exercise (27), and few studies have examined this practice in team sport settings (28–30). In female soccer players, CHO MR improved speed in short shuttle sprint performance (29). CHO MR did not affect repeated sprint performance in male soccer and rugby players (28), but it did prolong the onset of fatigue and reduce perceptions of effort in increasing-speed shuttle running to exhaustion in male lacrosse players (30). However, existing team sport research protocols are limited by the minimal inclusion of sport-specific exercises or scrimmages, which serve to replicate the rapid and sporadic changes in direction typical of training and competition (8). To date, there has been no CHO MR research in ice hockey.

Historically, measurement of ice hockey performance has been impaired by the difficulty of comparing off-ice to on-ice activity due to the prevalence of different surfaces (i.e., floor vs. ice), as well as the unique biomechanics facilitated by skating (31–33). The development of valid and reliable wearable technologies for indoor sports, such as local positioning systems (LPS) with Bluetooth heart rate (HR) monitors (34, 35), has enabled the collection of external and internal load data during on-ice training and competition (2, 3, 36).

External load is defined as the physical work performed during exercise and pertains to the organization, quality, and quantity of exercise (37). Examples of external load measures in team sports such as ice hockey include speed, total distance, and relative distance by speed zones, and explosive movements such as accelerations, decelerations, and changes in direction (2, 3, 5, 37, 38). Internal load represents the psychophysiological response to external load (36, 37). Existing measures of internal load in ice hockey include RPE, HR, and HR-derived training impulse (TRIMP), which combines exercise time, intensity and relative weighting of intensity (36, 39, 40).

Therefore, the present study used wearable LPS and HR microtechnology to examine the effects of MR with CHO vs. PLA solutions on external and internal loads in hydrated male ice hockey players during on-ice scrimmages with three regulation periods and one overtime (OT) period. It was hypothesized that relative to PLA, CHO MR would attenuate decreasing external load and increasing internal load: (1) as the 3 periods of regulation progressed, and (2) within the OT period.

## Materials and Methods

### Subjects

Twelve skilled male ice hockey players volunteered for this study (22.6 ± 3.4 years, 178.9 ± 4.7 cm, 84.0 ± 6.5 kg, skill range: AA U18–collegiate/major junior). Participants were active (~1.5 h/day, 5 days/week) and regularly played organized hockey. Participants were informed verbally and in writing about the study risks, before obtaining written consent. Ethical approval was obtained from the University of Guelph Research Ethics Board.

### Study Design

**Figure 1:**
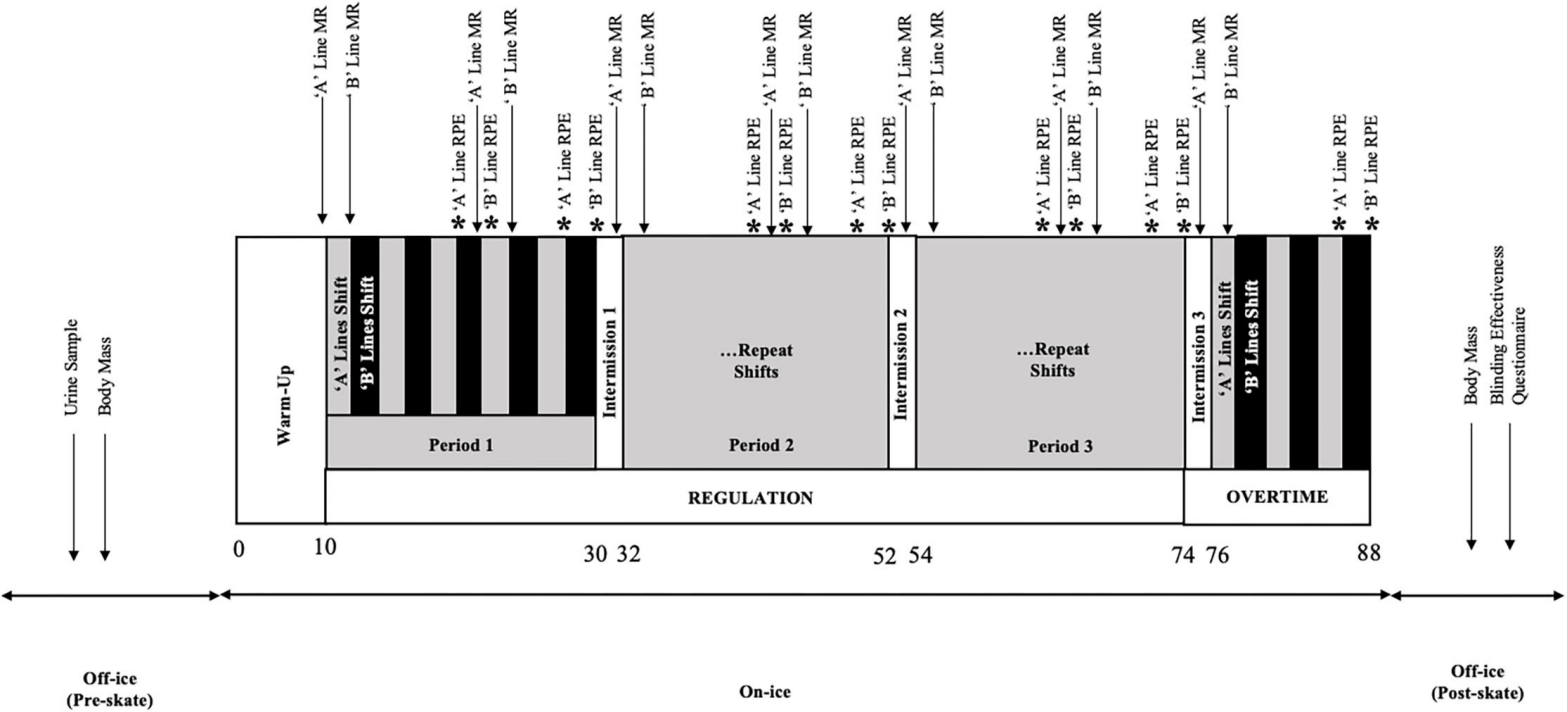
Experimental trial timeline. Numbers are in minutes. MR, mouth-rinsing; RPE, rating of perceived exertion.

This study had a double-blind, randomized, crossover design which included 1 familiarization trial and 4 experimental trials. Trials occurred at the same time (1:00–3:30 pm) and were a minimum of 48 h apart. Participants refrained from alcohol and strenuous exercise in the 24 h preceding each trial and were instructed to maintain the same dietary habits each trial day as they would in preparation for a game. Neither prescrimmage caffeine nor CHO intake was restricted. Pre- and post-skate measures were performed off-ice and included urine-specific gravity (USG) and body mass (BM) (Figure 1). USG was measured with a hand-held “pen” refractometer (ATAGO USA Inc., Bellevue, WA, USA), and values <1.020 were accepted as hydrated (17). BM was measured with participants in minimal clothing. Participants were on-ice for ~90 min, which included 10 min of warm-up, 3 20-min regulation periods, and one 12-min overtime period of small-sided 3-on-3 scrimmage with goaltenders, and 3 2-min intermissions. During the scrimmage, skaters hydrated with water and performed a 10-s MR approximately every 10 min with 25 mL of CES (Gatorade: 6% CHO, 19 mM sodium, 11 mM chloride, 3 mM potassium), or a noncaloric, taste- and electrolyte-matched placebo solution (PLA; Gatorade Zero). Presently, there is great inconsistency across the literature regarding solution concentration, length, frequency, and number of MRs (26, 27). The procedures used herein align with the most prevalent protocols to have displayed positive effects on high-intensity exercise performance.

Familiarization and experimental trials had the same structure, except familiarization included the additional protocol of measuring participant sweat loss. Skaters voided their bladder for the pre-skate urine sample. On-ice, skaters drank water *ad-libitum* from assigned bottles that were weighed pre- and post-skate. Sweat amount was equal to [(pre-BM – post-BM) + (fluid intake – urine output)]. This information was used to establish individual fluid intake parameters necessary to prevent mild dehydration in experimental trials. Within the latter, players were provided with two bottles with a volume of fluid equivalent to their expected sweat amount and were given frequent encouragement to drink, with the goal of finishing one bottle by the end of the second period, and the other at the end of OT.

### Methodology

#### On-Ice Scrimmages

**Figure 2:**
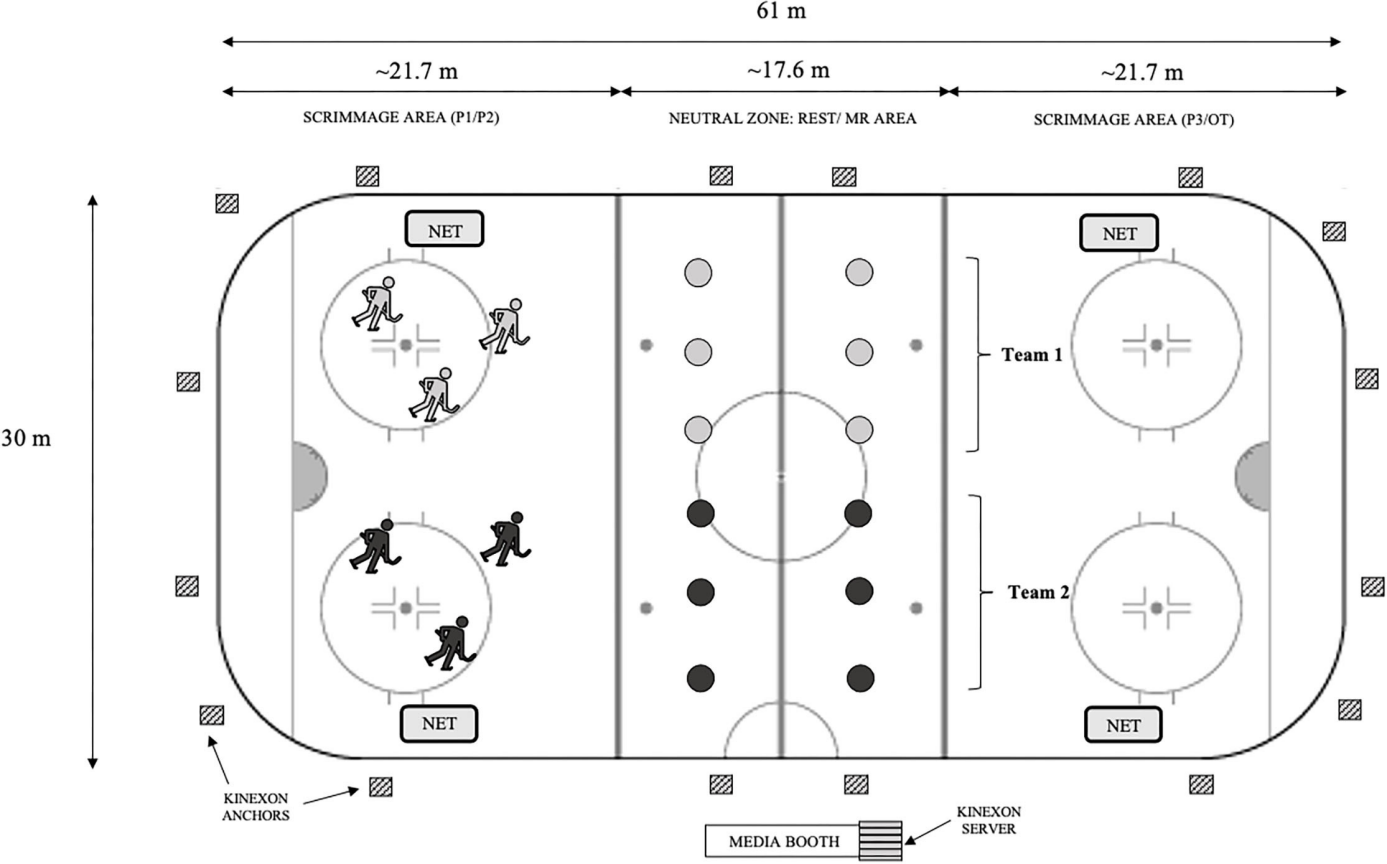
Schematic diagram outlining the ice-rink set up for small-sided 3-on-3 scrimmage and the location of one server and 16 anchors of an ultrawideband local positioning system (LPS; Kinexon, Munich, Germany) used to measure on-ice movements of skaters during scrimmage. Circles represent player rest/MR stations. MR, mouth-rinsing; P1, period one; P2, period two; P3, period three; OT, overtime.

Scrimmages were played across the width of an Olympic-sized rink (1.5 ± 0.9°C, 56.4 ± 4.2% humidity) between the blue line and end boards (Figure 2). Nets were set up ~2 m from the boards on the midline of the area. The space between the two blue lines (neutral zone) was used as a rest area for players. Research occurred during the COVID-19 pandemic, so a 3-on-3 scrimmage structure was adopted over 5-on-5 to abide by public health gathering guidelines. Each team had 6 players to permit rotation of 2 lines. A similar “small-sided game” set-up had previously been observed to elicit high-intensity game play which mimicked conventional 5-on-5 competition (41). The entire scrimmage included three 20-min regulation periods and one 12-min OT period, separated by 2-min intermissions. Regulation and OT were treated as separate events, as the score was always reset in the latter to imitate the tied score that would necessitate the additional “winner-take-all” OT period in a professional hockey competition (42). Within periods, skaters repeated 2-min shift and rest intervals, with changes made on a whistle. During the rest phases skaters remained seated. Lines were matched on the basis of skill to ensure fair gameplay (assessed during familiarization scrimmage and initial screening questionnaire). Six players with the highest skill level were put into group A and were randomly divided into two lines of 3 (“A Lines”). The remaining 6 players were put into group B and randomly divided into two groups of 3 (“B Lines”). The two lines in group A competed only against each other throughout the study as did the two lines in B. To encourage a high-level of competition, monetary awards were granted to players of the winning team for regulation and for OT.

#### MR Protocol

Mouth-rinse was performed at rest, preceding the first shift of every period (regulation and OT), and at roughly the mid-point of each regulation period, for a total of 7 rinses (Figure 1). Rinse aliquots were premeasured (25 mL) and administered orally *via* a plastic syringe. MR involved vigorous swishing of solution around the mouth for 10 s before expectorating the entire volume into a bucket. Participants were instructed to refrain from drinking any water in the remaining rest time after each rinse to prevent accidental ingestion of the solution. Individuals were assigned one MR condition per trial, and CHO and PLA conditions were randomized within teams and lines by a blinded researcher as to not confound scrimmage outcome. Duplicate experimental trials were completed for each condition. Blinding effectiveness was assessed *via* a postskate questionnaire that asked participants whether they felt they knew which rinse condition they had received. If the participants answered “yes” to this question, they were asked to identify the condition and explain their reasoning.

#### Athlete Monitoring

On-ice external loads were tracked using a tri-axial LPS (Kinexon Sports & Media, New York, NY, USA) which operated through specific local network access, one Power over Ethernet switch, one server, 16 anchors (secured to the arena rafters), and individual player sensors (Figure 2). Unpublished observations from our group have confirmed the validity and reliability of this system for use in ice hockey. Briefly, LPS variables including time on ice, skating distance, peak and average speed, and peak acceleration and deceleration demonstrated intersensor reliability (coefficient of variation (CV) <10%) when comparing two sensors worn by the same player. Also, when compared against a previously validated robotic sprint device (1080 Sprint, 1080 Motion, Lidingo, Sweden), near perfect correlations were observed between systems for instantaneous speed (*r* = 0.892, *p* < 0.001), peak speed (*r* = 0.989, *p* < 0.001), and acceleration (*r* = 0.968, *p* < 0.001), with very low CVs for all (<3%).

In the present study, sensors were secured to the exterior shoulder pads, positioned between the scapulae. LPS sensors included accelerometers to quantify linear motion in all directions, gyroscopes to measure angular motion and rotation, and magnetometers to measure direction and orientation of body position (44). To measure on-ice internal loads, players wore HR sensors (Polar OH1, Polar Electro OY, Kempele, Finland) secured in a band around the mid-bicep to avoid interference with protective equipment. These data were transmitted *via* Bluetooth to LPS sensors. Ultrawideband (UWB) channels (3244.88–4742.40 MHz) allowed for communication between LPS sensors and anchors, enabling real-time collection of data (20 Hz), transmitted to the server *via* hardwired connection. Data were stored on the LPS platform and could be retrieved on a secure computer for live or retrospective analysis. Commencement and cessation of data collection occurred automatically as players entered and exited the ice surface.

External load variables were derived from spatial and temporal LPS data and included distances, speeds, and explosive movements (accelerations, decelerations, sprints). Definitions of these metrics were adapted from One & Media—KNX ONE Hockey Metrics (Kinexon, Munich, Germany). Distance skated was categorized according to 6 speed zones: zone 1–very slow = 1.0–10.9 km·h^−1^, zone 2–slow = 11.0–13.9 km·h^−1^, zone 3–moderate = 14.0–16.9 km·h^−1^, zone 4–fast = 17.0–20.9 km·h^−1^, zone 5–very fast = 21.0–24.0 km·h^−1^, and zone 6–sprint = >24.0 km·h^−1^ (5). Distances traveled at low-intensity (<17 km·h^−1^) and high-intensity (≥17 km·h^−1^) speeds were also reported (3). Average and peak speeds (km·h^−1^) were determined from instantaneous changes in position and time. Accelerations were defined by positive rates of change of velocity (>2.0 m·s^−2^), while decelerations were defined by negative rates of change of velocity (<-2.0 m·s^−2^). A sprint was an action ≥22 km·h^−1^, maintained for at least 1 s.

Physiological indicators of internal load were measured across each period and included peak, average HR, and TRIMP (27, 45). RPE (Borg-10 scale) provided a psychophysiological measure of internal load and was recorded immediately following players' third and last shifts in periods 1–3, and the last shift in OT (Figure 1).

### Statistical Analysis

All participant data were from duplicate trials and were reported as mean (SD). Data from regulation and OT were treated as separate events. Data were confirmed to present a normal Gaussian distribution with a Shapiro–Wilk test. Data from regulation periods 1–3 were analyzed with two-way repeated measures ANOVAs (condition × period). When a significant *F* ratio was found, a *post-hoc* analysis was performed using Tukey's multiple comparisons test with adjusted *p*-values. Data collected in OT were analyzed using paired *t*-tests. Effect sizes (ES) were reported as partial η^2^ ($\eta_{p}^{2}$) for ANOVA (small = 0.01– 0.059, moderate = 0.06–0.139, large = ≥ 0.14) and Cohen's *d* for pairwise comparisons (small = 0.2–0.59, moderate = 0.6–1.19, large = 1.2–1.99, very large = ≥ 2.0) (45). All statistical analyses were performed with GraphPad Prism 9.1.0 for Mac (Graphpad Software, LLC, San Diego, CA). Significance was accepted at *p* ≤ 0.05, with confidence intervals (CI) of 95%.

## Results

### Blinding Effectiveness

There were 10/48 trials where players (*n* = 5) were confident they knew which MR solution they received. However, they were only correct 50% of the time, such that the correct MR solution was identified 5/48 times.

### Hydration Status

The average pre-scrimmage USG for all trials was 1.007 ± 0.006. Average player sweat loss was 1.38 ± 0.31 L and sweat rate was 0.97 ± 0.22 L·h^−1^. Participants maintained hydration during the CHO and PLA trials, losing only 0.32 ± 0.01 and 0.33 ± 0.01% BM, respectively.

### Performance in Regulation Play

#### External Load

There was no difference in total distance skated between MR conditions (CHO: 6,176 ± 287 m, PLA: 6,292 ± 402 m). For both conditions, there was a main effect of period [*F*_(2, 22)_ = 6.45, *p* = 0.006, $\eta_{p}^{2}$ = 0.71], and distance in period 1 (2,130 ± 19 m) was greater than periods 2 (2,062 ± 11 m, *p* = 0.008) and 3 (2,072 ± 29 m, *p* = 0.024).

**Figure 3:**
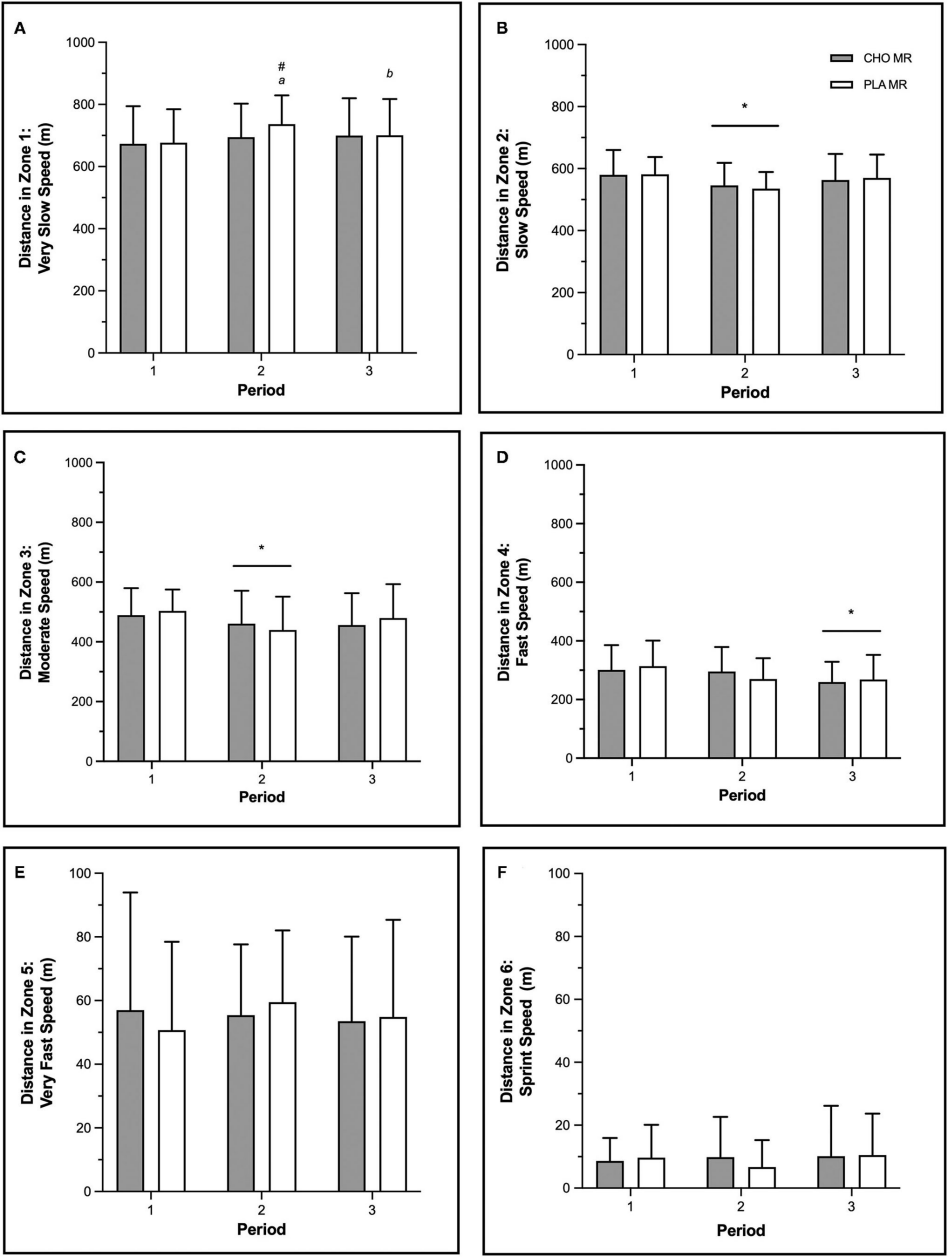
Distance traveled by male high-level hockey players (*n* = 12) in **(A)** zone 1–very slow (1.0–10.9 km·h^−1^), **(B)** zone 2–slow (11.0–13.9 km·h^−1^), **(C)** zone 3–moderate (14.0–16.9 km·h^−1^), **(D)** zone 4–fast (17.0–20.9 km·h^−1^), **(E)** zone 5–very fast (21.0–24.0 km·h^−1^), and **(F)** zone 6–sprint (>24.0 km·h^−1^) speed zones when mouth-rinsing with carbohydrate (CHO) or placebo (PLA) solution during three 20-min regulation periods of small-sided 3-on-3 ice hockey scrimmage. Data are mean (SD). *Main effect of period, significantly lower than period 1. ^a^Significantly greater than period 1, same condition. ^b^Significantly lower than period 2, same condition. ^#^Signifcantly greater than other MR condition, same period. Significance accepted at *p* ≤ 0.05.

In zone 1 there was a significant two-way interaction between period and condition [*F*_(2, 22)_ = 5.07, *p* = 0.015, $\eta_{p}^{2}$ = 0.46; Figure 3A]. Within period 2, significantly less distance was skated with CHO (695 ± 108 m) compared to PLA MR (737 ± 92 m, *p* = 0.005). For PLA MR only, significantly greater distance was skated in period 2 (737 ± 92 m) compared to period 1 (677 ± 108 m, *p* < 0.001) and period 3 (701 ± 116 m, *p* = 0.21). There was a main effect of period on distance in zones 2 [*F*_(2, 22)_ = 4.58, *p* = 0.022, $\eta_{p}^{2}$ = 0.93], 3 [*F*_(2, 22)_ = 3.58, *p* = 0.045, $\eta_{p}^{2}$ = 0.53], and 4 [*F*_(2, 22)_ = 9.15, *p* = 0.001, $\eta_{p}^{2}$ = 0.79]. Across both conditions, greater distance was skated in period 1 compared to period 2 in zones 2 (*p* = 0.018) and 3 (*p* = 0.037), and period 1 compared to period 3 in zone 4 (*p* < 0.001) (Figures 3B–D). There were no differences in the distances of zones 5 and 6 (Figures 3E,F).

There was a main effect of period for distance at high-intensity speed [*F*_(2, 22)_ = 6.04, *p* = 0.008, $\eta_{p}^{2}=$ 0.40], and the distance for both conditions in period 3 (328 ± 7 m) was less than period 1 (370 ± 5 m, *p* = 0.006). There was a main effect of period on average speed [*F*_(2, 22)_ = 5.73, *p* = 0.010, $\eta_{p}^{2}$ = 0.41], which decreased in both conditions from period 1 to periods 2 (*p* = 0.012) and 3 (*p* = 0.041) (Table 1).

**Table 1:** Speed and explosive movement external load variables of hydrated male high-level hockey players (*n* = 12) when mouth-rinsing with carbohydrate (CHO) or placebo (PLA) solution during three 20-min regulation periods of small-sided 3-on-3 ice hockey scrimmage.

**Variable**	**Condition**	**Period 1**	**Period 2**	**Period 3**
Peak speed (km·h^−1^)	*CHO MR*	24.3 (1.3)	24.2 (1.3)	24.4 (1.6)
	*PLA MR*	24.5 (1.2)	24.4 (1.4)	24.6 (1.7)
Average speed (km·h^−1^)	*CHO MR*	12.6 (0.9)	12.4 (0.9)^*^	12.3 (0.7)^*^
	*PLA MR*	12.8 (0.7)	12.3 (0.9)^*^	12.6 (1.0)^*^
Sprints (number)	*CHO MR*	2.7 (1.9)	2.5 (1.5)	2.4 (1.6)
	*PLA MR*	1.9 (1.1)	2.4 (1.1)	2.3 (1.2)
Peak acceleration (m·s^−2^)	*CHO MR*	3.5 (0.3)	3.4 (0.3)	3.3 (0.3)
	*PLA MR*	3.5 (0.2)	3.6 (0.2)	3.6 (0.2)
Accelerations (number)	*CHO MR*	12.8 (4.8)	12.0 (3.9)	11.5 (3.9)
	*PLA MR*	13.3 (4.6)	12.4 (3.7)	12.5 (4.4)
Peak deceleration (m·s^−2^)	*CHO MR*	4.7 (0.5)	4.6 (0.5)	4.4 (0.4)
	*PLA MR*	4.6 (0.4)	4.7 (0.3)	4.4 (0.5)
Decelerations (number)	*CHO MR*	19.3 (4.2)	15.6 (4.1)^*^	15.7 (3.8)^*^
	*PLA MR*	19.9 (5.9)	16.0 (3.5)^*^	15.8 (5.2)^*^

*A sprint is a movement at any speed ≥ 22 km·h^−1^, maintained for at least 1 s. Accelerations (>2.0 m·s^−2^). Decelerations (<−2.0 m·s^−2^). Values are presented as mean (SD)*.

* *Main effect of period, significantly lower than period 1 (p ≤ 0.05)*.

There was a main effect of period on number of decelerations [*F*_(2, 22)_ = 17.39, *p* < 0.001, $\eta_{p}^{2}$ = 1.93], which decreased in both conditions from period 1 to periods 2 and 3 (*p* < 0.001 for both) (Table 1).

#### Internal Load

Peak HR displayed a main effect of time [*F*_(2, 22)_ = 11.94, *p* < 0.001, $\eta_{p}^{2}=$ 0.79], and was lower in both conditions in period 1 (183.6 ± 1.2 bpm) compared to period 2 (187.5 ± 0.4 bpm, *p* < 0.001) and 3 (186.9 ± 1.7 bpm, *p* = 0.002). Average HR exhibited a significant two-way interaction between period and MR condition [*F*_(2, 22)_ = 5.09, *p* = 0.015, $\eta_{p}^{2}$ = 0.46], and within both conditions, period 1 (CHO: 147.8 ± 11.1 bpm, PLA: 145.6 ± 11.1 bpm) was lower than periods 2 (CHO: 155.3 ± 10.3 bpm, PLA: 156.9 ± 10.4 bpm, *p* < 0.001 for both) and 3 (CHO: 155.0 ± 10.9 bpm, PLA: 157.4 ± 11.3 bpm, *p* < 0.001 for both). Across regulation for CHO and PLA MR, peak HR values were 94 ± 4% and 94 ± 4% of age-predicted maximum and average HR vales were 77 ± 5% and 78 ± 6% of age-predicted maximum, respectively.

There was a significant two-way interaction between period and condition for TRIMP [*F*_(2, 22)_ = 4.11, *p* = 0.030, $\eta_{p}^{2}$ = 0.37]. Within CHO and PLA conditions, period 1 (CHO: 37 ± 11 AU; PLA: 35 ± 12 AU) was lower than periods 2 (CHO: 43 ± 13 AU, *p* = 0.002; PLA: 45 ± 12 AU, *p* < 0.001) and 3 (CHO: 42 ± 15 AU, *p* = 0.016, PLA: 46 ± 15 AU *p* < 0.001). TRIMP values across regulation were not different between conditions (CHO: 122 ± 37 AU, PLA: 126 ± 38 AU).

Ratings of perceived exertion displayed a main effect of period [*F*_(5, 55)_ = 8.28, *p* < 0.001, $\eta_{p}^{2}=$ 1.01], and in both conditions average values were lower in period 1 vs. 2 (*p* = 0.001) and 3 (*p* = 0.007). In periods 2 and 3 there was a non-significant trend for RPE to be lower with CHO (RPE values: period 1 = 6.7, period 2 = 7.2, and period 3 = 6.9) compared with PLA (RPE values: period 1 = 6.7, period 2 = 7.4, and period 3 = 7.4).

### Performance in Overtime Play

#### External Load

**Figure 4:**
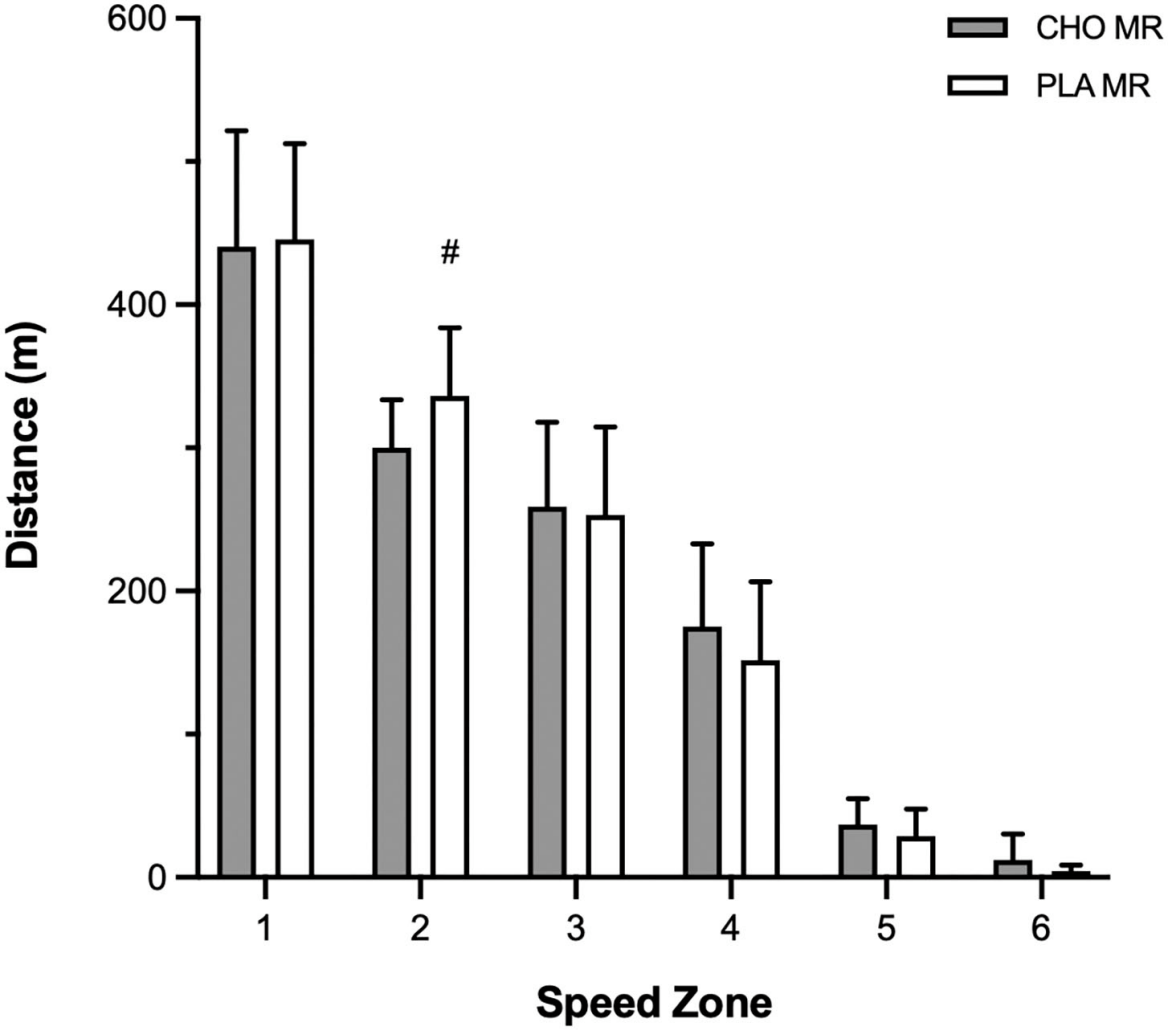
Distance traveled by male high-level hockey players (*n* = 12) in zone 1–very slow (1.0–10.9 km·h^−1^), zone 2–slow (11.0–13.9 km·h^−1^), zone 3–moderate (14.0–16.9 km·h^−1^), zone 4–fast (17.0–20.9 km·h^−1^), zone 5–very fast (21.0–24.0 km·h^−1^), and zone 6–sprint (>24.0 km·h^−1^) speed zones when mouth-rinsing (MR) with carbohydrate (CHO) or placebo (PLA) solution during one 12-min overtime period of small-sided 3-on-3 ice hockey scrimmage. Data are mean (SD). ^#^Signifcantly greater than other MR condition, same zone (*p* ≤ 0.05).

**Figure 5:**
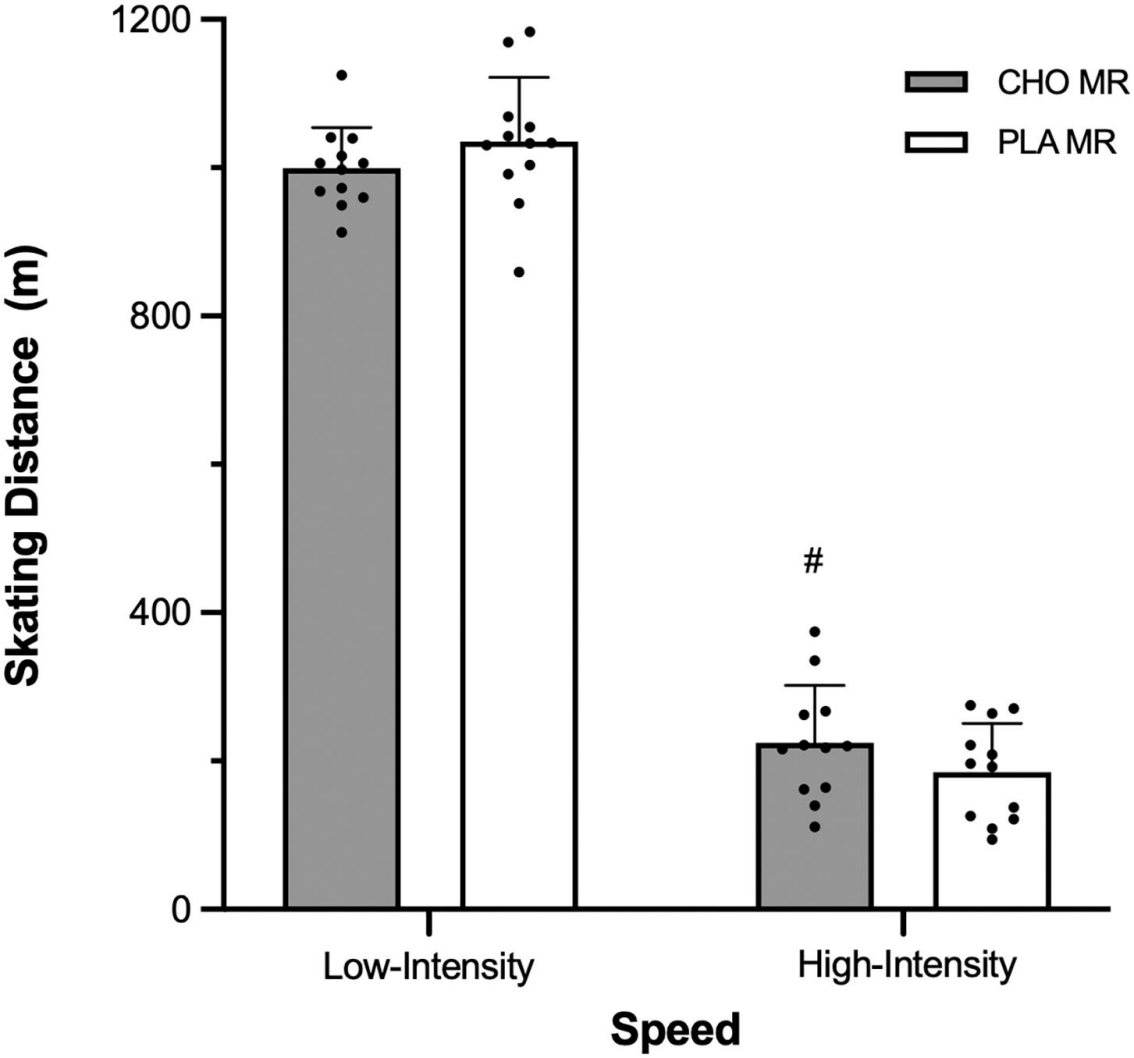
Distance traveled by male high-level hockey players (*n* = 12) at low-intensity (<17 km·h^−1^) and high-intensity (≥17 km·h^−1^) speeds when mouth-rinsing with carbohydrate (CHO) or placebo (PLA) solution during one 12-min overtime period of small-sided 3-on-3 ice hockey scrimmage. Data are mean (SD). ^#^Signifcantly greater than other MR condition, same speed (*p* ≤ 0.05).

There was no difference in total distance skated between CHO and PLA MR in OT (1,229 ± 78, 1,225 ± 99 m). In zone 2, greater distance was skated with PLA compared to CHO MR (*p* = 0.034, ES = 0.89; Figure 4). Distance skated was comparable between MR conditions in the other 5 speed zones. Distance at high-intensity speed was significantly greater with CHO relative to PLA MR (*p* = 0.042, ES = 0.54; Figure 5).

**Figure 6:**
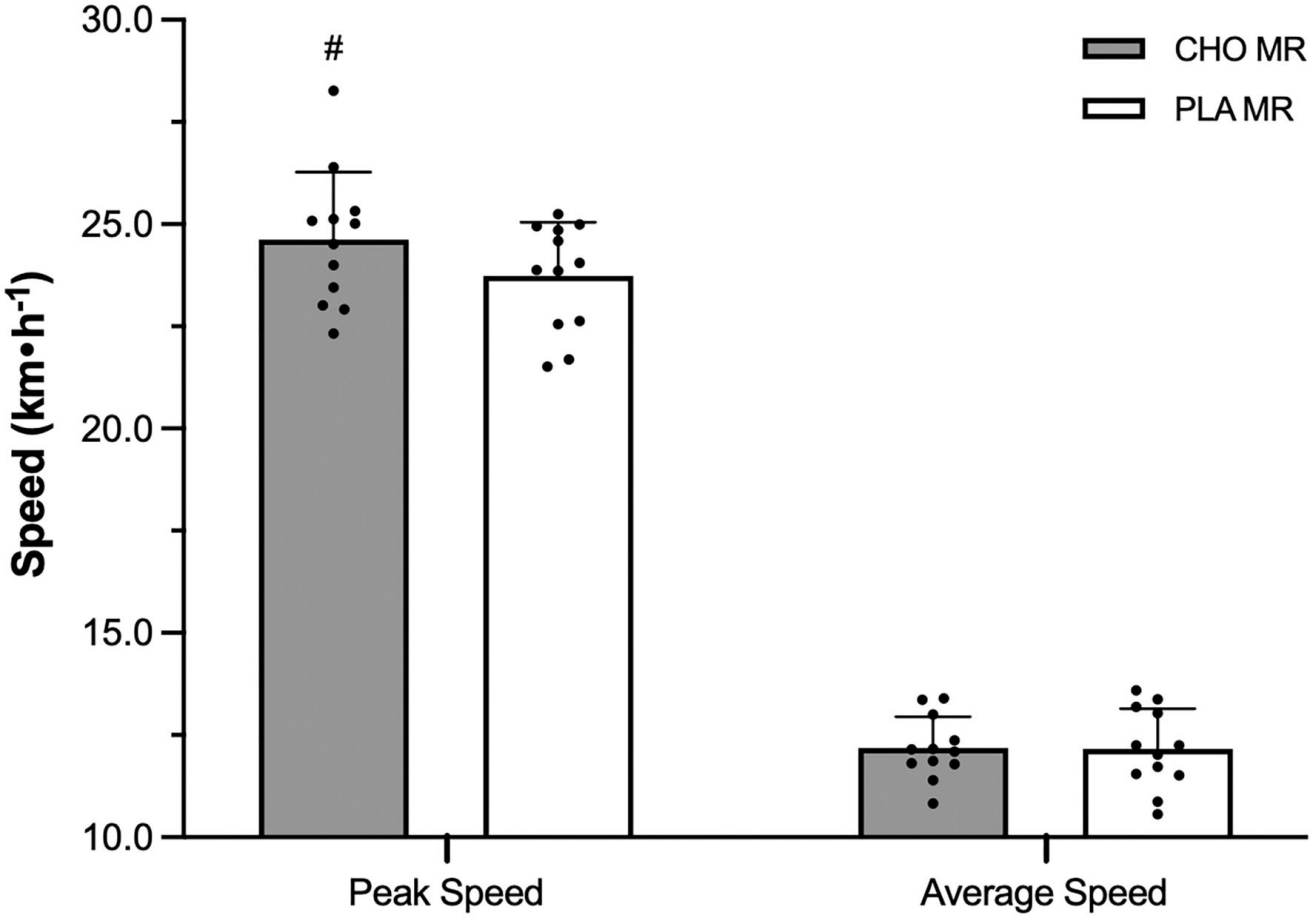
Peak and average speeds of male high-level hockey players when mouth-rinsing with carbohydrate (CHO) or placebo (PLA) solution during one 12-min overtime period of small-sided 3-on-3 ice hockey scrimmage. Data are mean (SD). ^#^Signifcantly greater than other MR condition, same variable (*p* ≤ 0.05).

Peak speed was higher with CHO over PLA MR (*p* = 0.016, ES = 0.62; Figure 6), but there was no difference in average speed between conditions. Average number of sprints performed was higher with CHO [1.9 ± 1.2] opposed to PLA [1.2 ± 0.9, *p* = 0.011, ES = 0.66]. There were no differences between CHO and PLA MR for number of accelerations (8.5 ± 3.5, 7.7 ± 2.1) and decelerations (9.7 ± 3.5, 10.3 ± 2.8), or peak acceleration (3.4 ± 0.4, 3.5 ± 0.4 m·s^−2^) and peak deceleration (−4.4 ± 0.5, −4.0 ± 0.5 m·s^−2^).

#### Internal Load

There were no differences between CHO or PLA MR for peak HR (183.3 ± 9.1, 185.7 ± 9.0 bpm), average HR (153.7 ± 11.8, 155.2 ± 11.3 bpm), TRIMP (24.9 ± 8.5, 26.4 ± 8.2 AU), or RPE (7.7 ± 0.9, 7.5 ± 0.9). For CHO and PLA MR, peak HR values were 93 ± 4% and 94 ± 4% for age-predicted maximum, and average HR values were 78 ± 6% and 79 ± 5% of age-predicted maximum, respectively.

## Discussion

This research examined the effects of CHO MR on external and internal loads in hydrated male ice hockey players during three regulation periods and one OT period of a small-sided 3-on-3 on-ice scrimmage. This is the first study to examine CHO MR in ice hockey, and the first MR research to use an entirely team sport-specific exercise protocol. The principle findings of this study were: (1) in regulation, there was a similar development of fatigue across both MR conditions from period 1 to 2 and 3, observed as decreases in external load metrics of total distance, high-intensity distance, number of decelerations, and average speed, and increases in internal load metrics of peak HR, average HR, TRIMP, and RPE, (2) CHO MR did not attenuate the decreases in external load and increases in internal load during regulation play, (3) in OT, peak speed, high-intensity distance, and number of sprints were greater with CHO over PLA MR, but (4) there were no differences in HR, TRIMP, or RPE between conditions in OT, despite elevated external loads with CHO MR. These results demonstrate that CHO MR improved physical performance in OT of an ice hockey scrimmage, and allowed athletes to perform more work relative to perceived levels of exertion.

### Regulation

There are limited recent descriptions of physical performance in ice hockey, and only two studies have included analysis of 3-on-3 play, despite its frequent occurrence in high-level competition (42). Lachaume and colleagues used small-sided 3-on-3 game, however the playing field was oriented differently, playing duration was shorter, the population was younger, and external loads were not monitored, severely limiting comparability to the present work (41). Alternatively, Lignell's group examined 3-on-3 full-ice competition, but only during an OT period (5). The present protocol has previously been used in other hockey research, but for analyzing goaltender performance (17). Therefore, the current analysis of player external and internal loads across time in small-sided 3-on-3 hockey scrimmage is novel.

The external loads observed herein were comparable to elite men's 5-on-5 full-ice hockey, where no change in distance was reported in the top speed zones between periods, but skaters displayed difficulty maintaining high-speed and performing explosive efforts across time (3, 5). Within the latter, marked skating fatigue was associated with the significant depletion of muscle glycogen, which occurred at the greatest rate in period 1 where players traveled ~900 m of high-intensity distance (3). During regulation in the present study, skaters traveled ~1,000 m of high-intensity distance, and accumulated similar total distance (~6,200 m) to 5-on-5 players (~6,000 m). Since anaerobic metabolism is the primary source of energy for high-intensity skating, transitions to higher speeds, and explosive efforts, it is proposed that late-scrimmage fatigue in small-sided 3-on-3 scrimmage was at least partly due to significant depletion of glycogen within skeletal muscle fibers (3, 14).

Average and peak HR values recorded in small-sided 3-on-3 scrimmage were similar to values reported in elite adolescent male small-sided 3-on-3 (41), but were slightly lower than university and elite men's 5-on-5 competition (3, 46). The lack of disparity between MR conditions is not surprising, as consumption of CES did not attenuate increases in HR in simulated or on-ice 5-on-5 scrimmage in recreational men (15, 16). Previous studies demonstrated reduced perceptions of fatigue with CES consumption in hockey, but no effects were observed presently. However, the former were compared to no-fluid trials, and therefore it is proposed that the maintenance of euhydration in the present protocol may have overpowered any additional benefit of oral CHO sensing on RPE in regulation. TRIMP values attained herein were greater than the values of university males in both regular season (95 ± 54 AU) and postseason (104 ± 68 AU) competitions (36). This is likely the result of far fewer players per team and increased active playing time in small-sided 3-on-3.

### Overtime

Despite similar total distance covered, players traveled lesser distance at slow speed, and more distance at high-intensity speed when MR with CHO was compared to PLA. Enhanced physical performance with CHO MR in OT is further highlighted by faster peak speeds and a greater number of sprints. Nonetheless, the external loads depicted within CHO and PLA MR conditions were both comparable to those reported in full-ice 3-on-3 OT by National Hockey League (NHL) players (5). The current players reached peak speeds (CHO MR: 24.6 ± 1.7 km·h^−1^, PLA MR: 23.7 ± 1.3 km·h^−1^) similar to NHL sprint speeds (24.5 ± 0.1 km·h^−1^), and actually traveled greater high-intensity distances [CHO MR: 224 ± 77 m, PLA MR: 185 ± 66 m] than NHL skaters [118 ± 17 m].

This is the first study to examine CHO MR in ice hockey, which adds to the paucity of information regarding MR and team sport performance. There are mixed findings within the existing literature, which provide limited evidence that CHO MR has small positive effects on speed and perceptions of fatigue in field sport athletes (28–30). The present findings exhibit the most favorable performance responses to CHO MR in intermittent high-intensity sport to date. Moreover, these results demonstrate that performance improvements observed in earlier hockey research with ingestion of exogenous CHO, as compared to no-fluid ingestion, may have been partially related to oral exposure to CHO (15–17).

For over a decade, the existence of oral receptors that respond to the energy content of CHO and increase the excitability of reward, motivation, and motor control brain regions has been known (21). Subsequent research demonstrated that oral sensing of CHO manipulated efferent outputs during voluntary contraction and task-specific actions, revealing positive physical responses to the perception of forthcoming energy (24, 25). Thus, it is proposed that during ice hockey, CHO MR might increase sensorimotor cortex activity and attenuate declines in motor function and neural drive to contracting muscles that are associated with fatigue, prolonging the quality of physical performance relative to the PLA condition. Notably, brain and sensorimotor responses to oral CHO exposure appeared to be greatest when energy status is low, which might explain why significant performance improvements in the present study were not observed until OT (24, 25).

Despite greater external loads with CHO MR, internal loads were not different between conditions. The absence of diverging RPE values between CHO and PLA MR with the development of physical fatigue opposes previous CHO-ingestion ice-hockey research (15–17). However, this observation has been frequently repeated in existing CHO MR exercise research (18–20). The present findings along with others demonstrate that instead of reducing perceptions of effort and fatigue, oral sensing of CHO permits maintenance of these perceptions despite greater work performed.

### Practical Applications

Carbohydrate MR is a simple, minimally disruptive practice that can be added to player nutrition and hydration regimes to facilitate heightened physical performance in situations where skaters are fatigued, such as OT. OT is a frequent occurrence in high-level hockey and is used to decide a winner when there is a tied score at the end of the three 20-min regulation periods (42). Within OT, the game is ended and won by the team that scores first. Most leagues have adopted a full-ice 3-on-3 structure, which facilitates increased space per skater and higher scoring chances. Therefore, the capacity to maintain speed and high-intensity actions in OT would be particularly advantageous.

This study demonstrated that CHO MR alone, without CHO ingestion, can produce beneficial effects on physical performance in hydrated male ice hockey players. Expectoration of MR solutions may be a favorable practice to attain the benefits of oral CHO exposure in athletes who prefer to consume minimal to no CHO during training and competition due to fear of experiencing gastrointestinal discomfort (47). However, swallowing after MR may be a more realistic practice for athletes in real-world competition. Beyond oral CHO exposure, drinking a CES throughout high-intensity sport can help prevent or alleviate the negative effects of dehydration by replenishing fluids and electrolytes lost through sweat (8). Ideally, carbohydrate supplementation practices should be customized based on individual preferences and designed to meet the needs of each athlete.

### Limitations and Future Directions

Strategic game-play in small-sided 3-on-3 scrimmage may have varied from 5-on-5 competition, which possibly altered external load variables (3, 5, 41). The smaller playing field may have forced players to rely more on technical skills vs. skating to generate offensive chances, and could have also prevented athletes from achieving true maximum speed. Together these factors may have reduced the distance and duration of high-intensity actions. Future research should replicate standard competition by including full-ice 5-on-5 play, followed by 3-on-3 OT, which could be shortened to 5 min (5). Adjustments to player work-to-rest ratios could further improve this protocol, as the current shift length (2 min) was longer than shifts in elite male 5-on-5 competition (30–80 s) (1–3, 5).

Though the present MR design was novel to ice hockey, future trial parameters should expand to include combinations of MR and ingestion. This would determine if previously observed performance benefits with CHO ingestion were due to oral exposure to CHO alone, or if there might be an additive effect with MR and ingestion (15–17). Additionally, special attention should be paid to nutritional control in forthcoming research, as it is possible that the absence of pre-trial nutritional restrictions in the present work may have impacted the findings herein.

Future research on ice hockey and MR would also benefit from the inclusion of female participants. Currently, there is one soccer MR study that included female participants (29), and a small number of general hockey studies that characterized female performance (38, 43). Therefore, a gap in the literature exists pertaining to the use of performance-enhancing strategies in female ice hockey players.

## Conclusions

This is the first study to examine CHO MR in ice hockey, and the only study to demonstrate multiple significant physical performance enhancements with CHO MR in a team sport-specific protocol. The positive effects of CHO MR on external loads were most prevalent with considerable development of fatigue. There were no effects in regulation, but in OT CHO MR improved physical performance, with less distance traveled at slow speed and increased distance traveled at high-intensity speeds, higher peak speed, and a greater number of sprints relative to PLA MR. It is proposed that these improvements were potentially due to manipulation of motor function stemming from alteration of central control through activation of oral CHO receptors.

Despite greater external load in OT with CHO MR, there were no differences in physiological or psychophysiological measures of internal load between conditions. This suggests that CHO MR allowed athletes to maintain perceptions of effort while performing relatively higher workloads. In conclusion, the results of this study indicate that CHO MR may be a valuable practice in ice hockey to protect against decrements in external load with increased playing time, and further, offers an alternative performance-enhancing solution for athletes who choose to avoid CHO ingestion during competition. Nonetheless, future work with CHO ingestion and MR trials would aid in understanding how to optimally administer exogenous CHO to enhance ice hockey performance.

## Data Availability Statement

The raw data supporting the conclusions of this article will be made available by the authors, without undue reservation.

## Ethics Statement

The studies involving human participants were reviewed and approved by the University of Guelph Research Ethics Board. The patients/participants provided their written informed consent to participate in this study.

## Author Contributions

DN and LS designed the study, interpreted the data, and wrote the manuscript. DN organized the trials and analyzed all data. DN, AG, JB, LB, AV, and LS collected on-ice data. AG and JB provided expertise with local positioning system data collection. All authors contributed substantially to revision and approved the final submission.

## Funding

Student support for Danielle Nyman was provided by a Queen Elizabeth II Graduate Scholarship in Science and Technology.

## Conflict of Interest

The authors declare that the research was conducted in the absence of any commercial or financial relationships that could be construed as a potential conflict of interest.

## Publisher's Note

All claims expressed in this article are solely those of the authors and do not necessarily represent those of their affiliated organizations, or those of the publisher, the editors and the reviewers. Any product that may be evaluated in this article, or claim that may be made by its manufacturer, is not guaranteed or endorsed by the publisher.
